# In-Situ Integration of 3D C-MEMS Microelectrodes with Bipolar Exfoliated Graphene for Label-Free Electrochemical Cancer Biomarkers Aptasensor

**DOI:** 10.3390/mi13010104

**Published:** 2022-01-09

**Authors:** Shahrzad Forouzanfar, Nezih Pala, Chunlei Wang

**Affiliations:** 1 Department of Bioengineering, Imperial College London, London SW7 2AZ, UK; s.forouzanfar@imperial.ac.uk; 2 Department of Electrical and Computer Engineering, Florida International University, Miami, FL 33174, USA; npala@fiu.edu; 3 Department of Mechanical and Materials Engineering, Florida International University, Miami, FL 33174, USA

**Keywords:** carbon, C-MEMS, graphene, bipolar exfoliation, cancer, aptamer, biosensor, label-free

## Abstract

The electrochemical label-free aptamer-based biosensors (also known as aptasensors) are highly suitable for point-of-care applications. The well-established C-MEMS (carbon microelectromechanical systems) platforms have distinguishing features which are highly suitable for biosensing applications such as low background noise, high capacitance, high stability when exposed to different physical/chemical treatments, biocompatibility, and good electrical conductivity. This study investigates the integration of bipolar exfoliated (BPE) reduced graphene oxide (rGO) with 3D C-MEMS microelectrodes for developing PDGF-BB (platelet-derived growth factor-BB) label-free aptasensors. A simple setup has been used for exfoliation, reduction, and deposition of rGO on the 3D C-MEMS microelectrodes based on the principle of bipolar electrochemistry of graphite in deionized water. The electrochemical bipolar exfoliation of rGO resolves the drawbacks of commonly applied methods for synthesis and deposition of rGO, such as requiring complicated and costly processes, excessive use of harsh chemicals, and complex subsequent deposition procedures. The PDGF-BB affinity aptamers were covalently immobilized by binding amino-tag terminated aptamers and rGO surfaces. The turn-off sensing strategy was implemented by measuring the areal capacitance from CV plots. The aptasensor showed a wide linear range of 1 pM–10 nM, high sensitivity of 3.09 mF cm^−2^ Logc^−1^ (unit of c, pM), and a low detection limit of 0.75 pM. This study demonstrated the successful and novel in-situ deposition of BPE-rGO on 3D C-MEMS microelectrodes. Considering the BPE technique’s simplicity and efficiency, along with the high potential of C-MEMS technology, this novel procedure is highly promising for developing high-performance graphene-based viable lab-on-chip and point-of-care cancer diagnosis technologies.

## 1. Introduction

The recent COVID-19 pandemic showed the importance of fast and on-site detection of pathogens in preventing and helping infected patients. The point-of-care (POC) biosensor can be used for fast and reliable detection of different pathogens and biomarkers on-site of patients. Likewise, the POC biosensors can be a powerful tool for early diagnosis and monitoring cancer disease patients. The largest benefit of POC sensing compared to conventional laboratory-based testing is that it can be done rapidly and be performed by clinical personnel who are not trained in clinical laboratory sciences. POC test results can provide a physician—and other clinical personnel—with answers that can quickly help determine a course of action or treatment for a patient [1,2]. Cancer diseases are among the main reasons for human death, and they kill millions of people every year [3]. The high fatality of most cancer diseases can be associated with the late diagnosis of these diseases, since the cancer patients show no physical symptoms until the late stages of the disease; however, the levels of associated cancer biomarkers change in early stages, which can be used for early diagnosis [4,5]. Among the discovered cancer biomarkers, platelet-derived growth factor-BB (PDGF-BB) is recognized to play a significant role in the growth and metastasis of several solid malignant tumors (e.g., breast [6], pancreatic [7], prostate [8], ovarian [9], and liver [10]), and its levels in the blood increase under the influence of the cancer diseases [11]. Hence, developing a POC biosensor for early diagnosis and monitoring the levels of PDGF-BB can be an immense help to many patients with cancer-associated solid tumors.

Electrochemical sensing is a highly suitable candidate for POC biosensing because of the possibility of label-free detection and miniaturization of electrochemical systems. Aptamer-based biosensors (also known as aptasensors) are a well-known type of electrochemical sensor in which aptamers are used for the detection of the bio targets (e.g., cancer biomarkers) [12,13]. Aptamers can provide several advantages over the other detection methods (e.g., enzymatic and immunosensing), including the high yield of fabrication, ease of manipulation (e.g., immobilization), low cost of synthesis, and high affinity and selectivity [4,14]. The suitability of electrochemical aptasensors for POC biosensing can be further explained by the fact that the cost of fabrication and operation can be considerably reduced. For instance, deploying novel synthesis techniques such as bipolar exfoliation can reduce the cost of fabrication [15,16,17,18]. Moreover, label-free electrochemical detection can reduce the cost of operation and blood sample consumption by requiring relatively simple sample preparation [19,20,21,22]; although, the performance of the proposed label-free electrochemical aptasensors (e.g., dynamic range, selectivity, and sensitivity) lags the performance of other sensing techniques such as optical and labeled aptasensors, which indicates the necessity of additional improvements.

Carbon-based microelectrochemical devices (C-MEMS) can be highly suitable for label-free electrochemical aptasensors. This class of carbon-based microdevices offers glass-like carbon’s unique features while providing a wide range of sizes (sub-micron to several microns) and various structures (e.g., 3-dimensional (3D) micropillars, nanowires, microneedles) [23]. The convenient features of glass-like carbon for label-free electrochemical biosensing include but are not limited to accessible surface modifications (e.g., increasing carboxyl groups sites for covalent immobilization of aptamers), a wide range of electrochemical stability (e.g., large voltage window and resilient toward corrosive agents), low background noise resulted from high conductivity and capacitance, and high tolerance against biofouling phenomenon [24,25,26,27]. One of the well-established techniques to fabricate C-MEMS devices is to synthesize glass-like carbon materials from SU-8 photoresist. This well-known and vastly investigated technique converts the developed SU-8 devices to glassy-carbon devices during a high-temperature treatment (typically between 800 °C to 1100 °C) in an oxygen-free environment. The synthesis parameters such as the heating ramp, the composition and the flow rate of the inert gas, and the final temperature can affect the properties of the developed C-MEMS-based devices. These parameters can be exploited to match better the intended application (e.g., tuning the pore size, shrinkage percentage, and percentage of carbon in the final product) [23,28]. Furthermore, C-MEMS technology enables manageable geometry manipulations in which they can be used to increase the physical surface area of the electrode (e.g., fabricating highly dense 3D micropillars). Increasing the physical surface area is an important aspect of developing every electrochemical biosensor type [3,24].

Graphene is a well-known 2-dimensional (2D) material in biosensing because of its unique properties, which can considerably enhance the performance of the developed biosensor [29]. Providing large surface area, high charge mobility, and eccentric superconductivity of graphene made this material a potent candidate for the development of biosensors [30,31]. However, some limitations exist in traditional graphene synthesis and implantation techniques, which can hinder the development of mass-producible POC biosensors. The issues such as huge variations of graphene contents in commercial products [32], the burdensome and toxic procedures of synthesis of graphene and reduced graphene oxide (rGO) [33], the complex and lengthy process of functionalization and deposition of graphene [34,35] can be considered as the limitations of traditional techniques. These issues can be effectively addressed by using novel bipolar exfoliation (BPE) of graphene technique. BPE is an environmentally friendly technique that can exfoliate, reduce, and deposit rGO on the desired electrode. Besides, Khakpour et al. have established that the BPE-rGO has vertically aligned morphology, eradicating the need to use spacers such as carbon nanotubes to prevent the aggregation of graphene nanoflakes [36,37].

This study investigated the in-situ integration of bipolar exfoliated rGO (BPE-rGO) on three-dimensional (3D) C-MEMS microelectrodes for the first time. This research aimed to explore the compatibility of the BPE technique with C-MEMS technology. Hence, the 3D C-MEMS microelectrodes were used as negative feeding electrodes in a BPE cell, and the deposition uniformity was investigated via scanning electron microscopy (SEM). Cancer aptasensors were developed based on the covalently immobilized amino-terminated ssDNA aptamers on the surface of the 3D C-MEMS microelectrodes decorated with BPE-rGO to explore the suitability of this novel electrode for biosensing application. The characteristics of the fabricated aptasensors were studied using Fourier-transform infrared spectroscopy (FTIR). Electrochemical and sensing performance analyses were conducted using cyclic voltammetry (CV). The results revealed that the integration of BPE-rGO with 3D C-MEMS enhances the areal capacitance of the microelectrodes and improves the sensitivity of the aptasensors.

## 2. Materials and Methods

### 2.1. Materials and Reagents

The PDGF-BB binding aptamer (ssDNA, amino linker-5′-C_6_-CAG GCT ACG GCA CGT AGA GCA TCA CCA TGA TCC TG-3′ [14]) was purchased in HPLC purification from ThermoFisher Scientific, Waltham, MA, USA. Tris-ethylenediaminetetraacetic acid (TE) buffer, ethanol, acetone, phosphate-buffered saline (1 M and pH 7.4) (PBS), 1-ethyl-3-(3-dimethylaminopropyl) carbodiimide hydrochloride linker (EDC), N-hydroxysuccinimide linker (NHS), hydrochloric acid (HCl), Polyoxyethylene (20) sorbitan monolaurate (Tween-20), KCl, and k_3_Fe(CN)_6_ were purchased from ThermoFisher Scientific, Waltham, MA, USA. Trehalose, bovine serum albumin (BSA), and platelet-derived growth factor-AA, AB, and BB were purchased from Sigma Aldrich, St. Louis, MO, USA. NANO^TM^ SU-8 25 and SU-8 100 negative photoresist were purchased from Microchem., Round Rock, TX, USA. Graphite rods (3 cm in length and 6.15 mm in diameter, Ultra “F” Purity 99.9995%) were purchased from Fisher Scientific, Rockingham County, NH, USA. The 316 stainless steel (SS) electrodes were purchased from Maudlin Inc., Kemah, TX, USA. All chemicals were analytical grade. Milli-Q (Sigma Aldrich, St. Louis, MO, USA) deionized water (DI water) was used in this study.

### 2.2. Apparatus

A Bio-Logic versatile multichannel potentiostat (VMP3) was used for the electrochemical analysis. JEOL SEM 6330 was used to obtain the SEM images of the electrodes. Agilent Technologies N6705A dc Power Analyzer applied a direct current (DC) voltage of 45 V across the feeding electrodes. An Ag/AgCl (KCl saturated) and Pt wire were used as the reference electrode and the working electrode in all electrochemical measurements, respectively. A 713 Metrohm pH meter was used for measuring the pH of electrolytes. JASCO FTIR 4100 was used to conduct FTIR analysis.

### 2.3. 3D C-MEMS Photolithography

**Figure 1:**
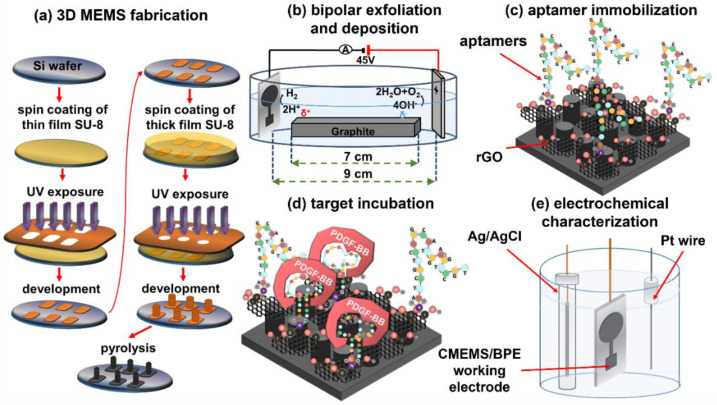
Schematic illustration of (**a**) 3D C-MEMS microfabrication process, (**b**) bipolar exfoliation cell, (**c**) development of C-MEMS/BPE/Apt PDGF-BB aptasensors, (**d**) target incubation, and (**e**) three-electrode electrochemical cell with Ag/AgCl reference electrode, Pt wire counter electrode, and C-MEMS/BPE/Apt in 5 mL aqueous electrolytes of 0.1 M PBS/5 mM K_3_Fe(CN)_6_/0.2 M KCl.

The 3D C-MEMS microelectrodes were fabricated via the previously reported C-MEMS synthesis process [3,14,36,37], schematically illustrated in Figure 1a. Briefly, the fabrication process includes producing a uniform layer of SU-8 25 with the thickness of 15 µm on a 4″ N-doped silicon wafer via spinning at 3000 rpm for 30 s, performing pre-exposure bakes at the recommended temperature, UV exposure with a dose of 300 mJ cm^−2^, and post-exposure bakes. After developing the first layer, the thick layer of SU-8 100 photoresist was spin-coated on the wafer at 300 rpm for 12 s and then accelerated to 2000 rpm for 30 s to provide a uniform photoresist layer with a thickness of approximately 150 µm. Soft bakes at 65 °C for 20 min and 95 °C for 60 min were conducted on hotplates, followed by UV light exposure with a dose of 700 mJ cm^−2^. On hotplates, post-exposure bakes were conducted at 65 °C for 2 min and 95 °C for 15 min. The developed electrodes were pyrolyzed in a Lindenberg tube furnace with a temperature ramp of 10 °C min^−1^ in two steps of 250 °C with a dwell time of 30 min, and 900 °C with a dwell time of 60 min, under 500 sccm flow of forming gas (5%H_2_ + 95%N_2_). The samples were left in the furnace overnight to cool to room temperature.

### 2.4. Bipolar Exfoliation and Deposition of rGO on 3D C-MEMS Microelectrodes

A bipolar exfoliation cell, illustrated in Figure 1b, has been used for exfoliation, reduction, and deposition of graphene nanosheets on 3D C-MEMS electrodes. The bipolar mechanism details have been discussed in previous studies [34,35,36]. Briefly, a 45 V DC voltage was applied through the C-MEMS microelectrode as the negative feeding electrode and 2 × 2 cm stainless steel positive feeding electrodes with a 9 cm distance to induce an electric field of 5 V cm^−1^. The resistivity of DI water used for bipolar exfoliation was 17 MΩ. After 24 h of bipolar treatment, the 3D C-MEMS electrodes decorated with BPE-rGO (designated as C-MEMS/rGO) were thoroughly washed in DI water and dried in an oven at 70 °C overnight to evaporate remaining moisture from C-MEMS/rGO electrodes.

### 2.5. Aptasensor Development

Previous studies have thoroughly discussed the deployed aptasensor development [14,15]. The process includes activating the amino-tags of aptamers in a solution of 20 mg mL^−1^ EDC and 10 µL of 20 mg mL^−1^ NHS and immobilizing the aptamers via the drop-casting method. After 2 h of incubation, electrodes were washed thoroughly in DI water to wash away freestanding aptamers. The remaining blank areas were covered with the aqueous solution of 0.1 M PBS + 1% (*v*:*v*) Tween-20 to reduce the non-selective physical adsorption of interference agents. The aptamer immobilized electrodes (designated as C-MEMS/rGO/Apt) (Figure 1c) were washed with DI water for 5 min and stored in 0.1 M TE buffer in the refrigerator (4 °C) when not in use.

### 2.6. Electrochemical Characterization of Aptasensors

The target detection process was initiated by incubating the sensing electrode with the desired target concentration at 30 °C for 40 min. These parameters were optimum temperature and duration for target detection based on the designed aptamers and electrodes [3,8]. Following the target entrapment illustrated in Figure 1d, the sensing electrodes were washed in DI water before electrochemical measurements. The sensing electrodes incubated with the target were immediately tested via the three-electrode setup illustrated in Figure 1e for CV evaluations. The 5 mL aqueous electrolyte was used for electrochemical measurements, based on a mixture of 5 mM k_3_Fe(CN)_6_, 0.1 M PBS, and 0.2 M KCl. In CV measurements, the electrodes were tested between −0.3 V to 0.6 V versus the reference electrode with a scan rate of 40 mV s^−1^. All CV data were typically taken after conducting 10 continuous cycles to ensure a reliable data recording. After every electrochemical measurement, the C-MEMS/rGO/Apt aptasensors were regenerated by immersing the sensing electrodes in 1 M TE buffer with gentle stirring for 30 min. The aptasensors were immersed in 0.1 M TE buffer and kept in a refrigerator (4 °C). Previous studies confirmed that the electrodes could remain active for 6 days [14,15].

## 3. Results and Discussion

### 3.1. Material Characterization

**Figure 2:**
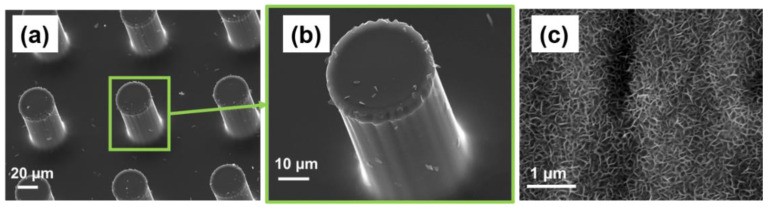
SEM images of (**a**), (**b**) C-MEMS micro pillars before BPE procedure, (**c**) BPE-rGO deposition on stainless steel control sample.

The C-MEMS technology enables the production of various geometries with a wide range of sizes. In this study, 3D C-MEMS microelectrodes consisted of a thin current collector electrode decorated with micropillars with a radius of 25 µm and a height of approximately 100 µm. The SEM image of the fabricated 3D C-MEMS microelectrodes is presented in Figure 2a,b. As it can be seen, the pillars are well defined, and no crack or curvature can be detected from the SEM images, which indicates that the C-MEMS technology is highly feasible for fabricating 3D high-aspect-ratio microstructures.

The previous study on the application of BPE technology has proven that synthesized rGO via BPE technique has high quality and is suitable for cancer aptasensors. That study showed that the graphene deposited on the negative feeding electrode has better properties for biosensing applications, including a porous morphology and higher areal capacitance [15]. Hence, the C-MEMS 3D microelectrodes were used as the negative feeding electrode in the BPE procedure. Figure 2c provides an SEM image of the BPE-rGO after 24 h of deposition on a stainless electrode (as a control sample) which a porous morphology can be identified, and the pore size of 100 nm can be estimated. Hence, it was hypothesized that the successful in-situ integration of BPE and C-MEMS techniques would produce similar deposition morphology on the C-MEMS microelectrode.

**Figure 3:**
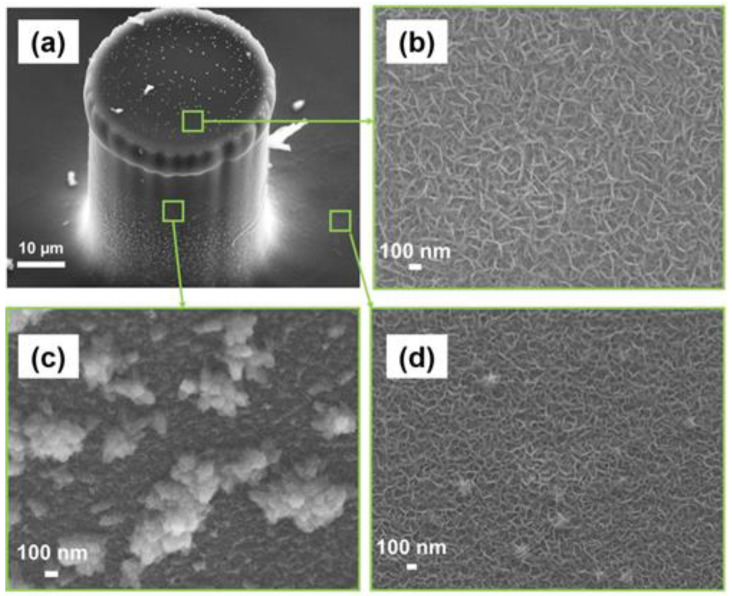
SEM images of (**a**) C-MEMS micro pillars with BPE-rGO, (**b**) BPE-rGO deposition on top of the micropillar, (**c**) BPE-rGO deposited on sides of the micropillar, and (**d**) BPE-rGO deposited on thin film base electrode.

The SEM image of the 3D C-MEMS micropillar covered with rGO deposition after 24 h of deposition is presented in Figure 3a. The SEM images of rGO deposited on top of the micropillar (Figure 3b), side of the pillars (Figure 3c), and the current collector electrode (Figure 3d) show that the deposition of BPE-rGO is uniform with a porous morphology similar to the control sample with a pore size of around 100 nm and vertically aligned structures. Hence, it can be concluded that BPE technology can deposit nano-porous rGO directly on C-MEMS microelectrodes with high uniformity and coverage without requiring spacers such as carbon nanotubes to prevent the agglomeration of rGO nanosheets.

**Figure 4:**
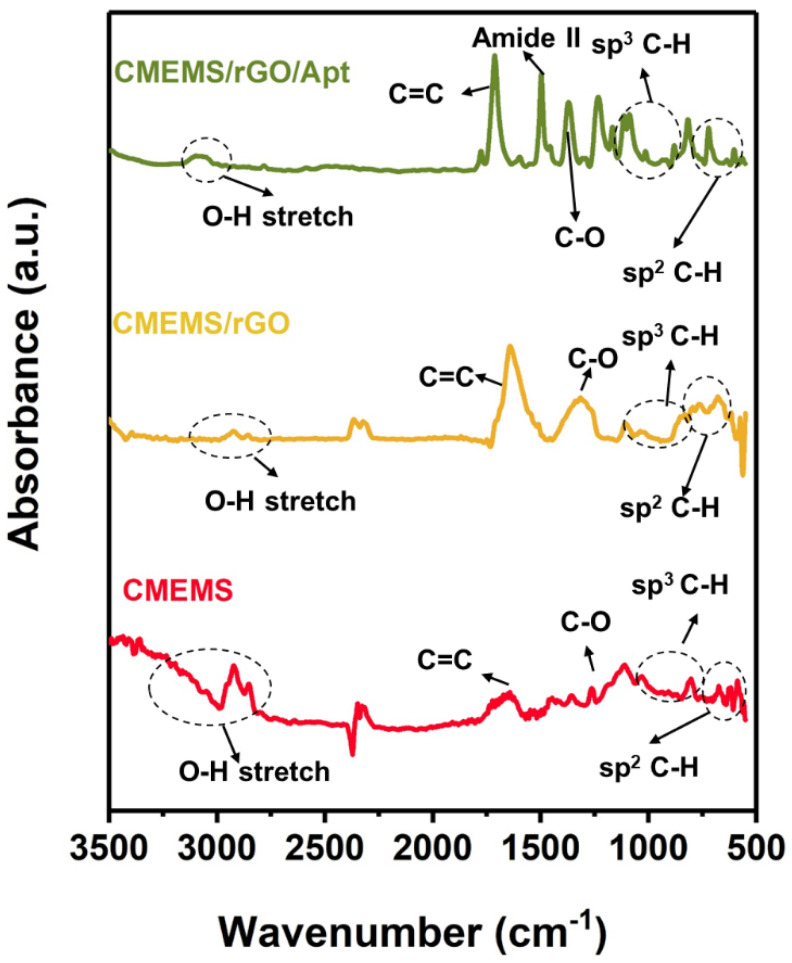
FTIR spectra ofCMEMS electrode before any modification, CMEMS electrode after BPE-rGO deposition (CMEMS/rGO), and CMEMS/rGO after aptamer immobilization (CMEMS/rGO/Apt).

In the previous study on BPE-rGO based biosensors, the achieved FTIR results showed that the BPE-rGO was partially reduced, and it contained a sufficient percentage of carboxyl groups to provide adequate binding sites for covalent immobilization of amino-terminated affinity aptamers [14,15]. Hence, it was hypothesized that a similar surface composition would be achieved for integrated BPE-rGO with C-MEMS 3D microelectrodes. Consequently, the FTIR characterization was conducted on electrodes in different stages of development, including C-MEMS microelectrodes before BPE treatment (designated as C-MEMS), after BPE-rGO deposition (C-MEMS/rGO), and after aptamer immobilization (C-MEMS/rGO/Apt). The FTIR spectra of C-MEMS, C-MEMS/rGO, and C-MEMS/rGO/Apt microelectrodes are represented in Figure 4. In all three studied samples, several peaks can be identified, which can be described as follows; a broad peak between 280 and 200 cm^−1^ ascribed to O-H stretching, peaks at 1100–1300 cm^−1^ attributed to sp^3^ C-H bending, and most noteworthy peaks at 1430 and 1600 cm^−1^ ascribed to C-O bending and aromatic C≡C stretching [36]. The FTIR spectrum for C-MEMS/rGO/Apt confirms a peak at 1571 cm^−1^, associated with amide II band, representing covalent bonding of PDGF-BB aptamers with the surface of C-MEMS/rGO/Apt microelectrodes [36]. The detected peak at 1571 cm^−1^ in FTIR spectra confirms that the locally formed carboxyl groups on the graphene surfaces can be used directly to immobilize the amino-terminated affinity aptamers. Although, the immobilization efficiency can be improved using oxygen–plasma etching to increase the percentages of carboxyl groups on the surface of the C-MEMS/rGO microelectrodes [3,36].

### 3.2. Electrochemical Characterization

**Figure 5:**
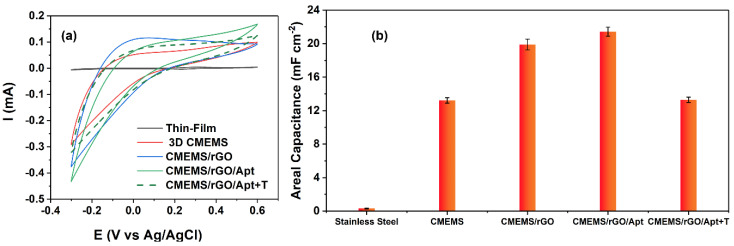
(**a**) CV plot of C-MEMS bare electrode and after each modification with scan rate of 40 mV s^−1^ and incubation with 100 pM PDGF-BB (**b**) Areal capacitances calculated from CV plots.

Several studies have shown that the deposition of rGO via the BPE technique on the perspective electrode considerably increases the electrode’s double layer (i.e., areal) capacitance [15,37,38]. For instance, Forouzanfar et al. have shown that the deposition of BPE-rGO on 0.5 cm^2^ stainless steel electrodes increases the areal capacitance from 0.80 mF cm^−2^ for the bare stainless steel electrodes to 2.69 mF cm^−2^ for the stainless steel electrodes with a thin layer of rGO [15]. Such increase in capacitance is a highly preferred aspect for label-free electrochemical biosensors since it can reduce the background noise and enhance the prospective biosensor’s sensitivity and limit of detection. Furthermore, the previous studies on C-MEMS technology have shown that the C-MEMS microelectrodes have large double-layer capacitances without any modifications, which can be promptly harvested for label-free electrochemical aptasensors [3,14]. Therefore, a compatible in-situ integration of C-MEMS and BPE deposition was expected to produce electrodes with sufficiently high capacitances for label-free detection of PDGF-BB biomarkers. In order to explore this hypothesis, CV tests were done on a stainless steel control sample and 3D C-MEMS microelectrodes with the same active area of 0.5 cm^2^ at each development stage. The CV analysis is given in Figure 5a. The area under the CV curve can be used to calculate the areal capacitance using Equation (1) [39]:

(1) $$C=\frac{1}{2\mathrm{As}\Delta V}\;\int^{\;}IdV$$

where Δ*V* is the voltage window, *s* is scan rate, *A* is the electrode area, *V* is the voltage, and *I* is the current. The calculated areal capacitances from CV curves are given in Figure 5b. The area under the CV curve of the 3D C-MEMS electrode was much larger than the CV curve of the stainless steel electrode, in which the increased area under the CV curve can be interpreted as increased areal capacitance from 0.32 mF cm^−2^ to 7.67 mF cm^−2^ for stainless steel electrodes and 3D C-MEMS microelectrodes, respectively. The increase in the capacitance can be explained by the increase in the surface area of the electrode and the better electrochemical properties of C-MEMS materials. After deposition of BPE-rGO, the areal capacitance was increased to 19.89 mF cm^−2^, resulting from the increased active area. These results confirm the earlier hypothesis regarding the capacitance enhancement of CMEMS/rGO compared to the bare C-MEMS microelectrode.

After immobilizing the aptamers, the areal capacitance was slightly increased to 21.43 mF cm^−2^, which could result from the decrease in the percentage of negatively charged species on the surface of the electrode due to the participation of negatively charged carboxylate groups of the surface in amide bonding (i.e., covalent immobilization of aptamers) and extinguished not-involved carboxyl groups by Tween-20 quencher. The previous study proved that entrapment of the PDGF-BB molecules diminishes the areal capacitance [14]. Therefore, a successful aptasensor was expected to show a declining capacitance in response to incubation with PDGF-BB. The calculated areal capacitances show a definitive decrease from 21.43 mF cm^−2^ to 13.29 mF cm^−2^ after incubation of the C-MEMS/rGO/Apt biosensor with 100 pM of target molecules. Hence, the developed label-free aptasensor successfully detected the target in a turn-off response fashion. The decrease in capacitance can be the consequence of two phenomena occurring during and after target entrapment. The first phenomenon is the result of the changes in helix structure of the affinity aptamers, which can make them closer to the surface of the sensing electrode, repel the negatively charged species, and disturb the double layer capacitance since aptamer are negatively charged [14,40]. The second phenomenon is the lessened passway of charge exchange between the electrolyte and electrode because of the PDGF-BB oncoproteins’ isolative properties [41].

### 3.3. Sensing Characterization

**Figure 6:**
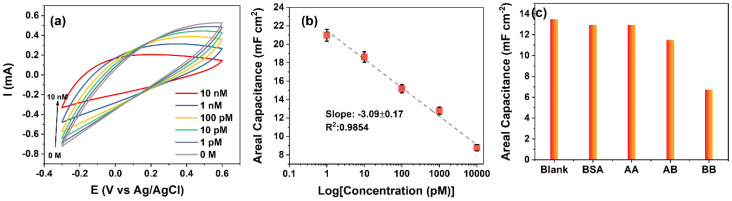
(**a**) CV curves of the C-MEMS/rGO/Apt electrode’s response to PDGF-BB ranging from 0–10 nM. (**b**) Calibration curve for calculated areal capacitances from CV curves with *n* = 3. (**c**) Areal capacitances calculated from CV curves measured in response of C-MEMS/rGO/Apt electrodes to 4 µg mL^−1^ BSA. 50 nM PDGF-AA, 50 nM PDGF-AB, and 500 pM PDGF-BB.

The electrochemical analysis confirmed that the novel in-situ deposition of rGO via BPE technique on C-MEMS 3D microelectrodes can produce a highly suitable sensing electrode that can detect the PDGF-BB biomolecules in a turn-off sensing mechanism (i.e., the increase in the target concentration leads to a decrease in output signal intensity or amount). Therefore, the capacitive response CMEMS/rGO/Apt electrodes to 0–10 nM PDGF-BB were collected (results are presented in Figure 6a to define the sensitivity and limit of detection of the aptasensor. The oxidation of peak Fe(CN)_6_^−3/−4^ at E = 0.3 V (vs. Ag/AgCl) was decreased proportionally to the increase in the PDGF-BB concentration. The observed decrease in the peak current results from the increased charge transfer resistance of the sensing electrode. The calibration curve for calculated areal capacitances using Equation (1) is shown in Figure 6b. The calibration curve was obtained by calculating the average value of areal capacitances (mF cm^−2^) from the repeated measurements of *n* = 3. The area under the CV curves decreased substantially in a linear manner upon increasing the PDGF-BB concentrations. The linear dependence of the output capacitance on the logarithm of PDGF-BB concentration has a slope of −3.09 mF cm^−2^ Logc^−1^ (unit of c, pM) and the R^2^ of 0.9854. The correlation of CMEMS/rGO/Apt capacitance to the logarithm of PDGF-BB concentration can be assessed as follows (Equation (2)),

C = 21.47 − 3.09 Logc^−1^, r = 0.9887 (2)

where C is the areal capacitance (mF cm^−^^2^), c is the PDGF-BB concentration (pM) and r is the regression coefficient. Hence, the CV measurement’s sensitivity to PDGF-BB can be calculated as 3.09 mF cm^−2^ Logc^−1^. The limit of detection (LoD) for CMEMS/rGO/Apt aptasensors based on CV measurements was calculated as 0.75 pM based on the linear regression (Equation (3)) [42],

(3) $$\mathrm{LoD}=\frac{3S}{b}$$

where S is the standard deviation of the blank response (STDEV = 0.0347 mF cm^−2^, *n* = 9), and b is the slope of the calibration curve (b = −13.88 mF cm^−2^ c^−1^ of areal capacitances). The low LOD and linear decrease in areal capacitance illustrate the two important features of the envisioned sensor. First, the developed sensing system is highly responsive to the changes in the surface of the electrode (i.e., the entrapment of the target molecules), and second, the sensing electrode material was capable of providing sufficient active sites for immobilizing aptamers which can be seen from the linearity and range of the sensors’ response to the target molecules.

The selectivity of the CMEMS/rGO/Apt aptasensors was studied by measuring the response of the aptasensors to 4 µg mL^−1^ BSA, 50 nM PDGF-AB, and 50 nM PDGF-AA along with 500 pM PDGF-BB. The concentrations of interference agents were chosen to be approximately 100 times more than that of the target molecules. The selectivity measurements are presented in Figure 6c, in which the areal capacitances were calculated from the CV curves. The change in capacitive response (i.e., the blank response capacitance minus the capacitance in response to the biomarker) of CMEMS/rGO/Apt aptasensors to PDGF-BB (a change in capacitance of −6.72 mF cm^−2^) was 12.73 and 3.34 times higher than that of the aptasensors to PDGF-AA (a change in capacitance of −0.54 mF cm^−2^) and PDGF-AB (a change in capacitance of −1.99 mF cm^−2^), respectively. The results indicate that the novel aptasensors could provide high sensitivity and sufficient selectivity due to the large active surface area of the BPE-rGO, fast electrochemical kinetics of the graphene, and high affinity of the aptamers.

Table 1 summarizes the recently developed electrochemical aptasensors. The current aptasensor exhibited a wide linear range. The linear response range of envisioned aptasensor based on CMEMS/rGO covers the healthy levels of PDGF-BB as well as elevated levels in patients with cancer diseases [43,44]. It is worth noting that the attained LoD of detection of 0.75 pM (the equivalent of 1.49 pg mL^−1^) is adequately lower than the minimum cut-off point of 0.1 ng mL^−1^ for the healthy levels of PDGF-BB in human serums [45]. In general, the proposed aptasensor provides a wide linear range with efficient accuracy (i.e., the limit of detection) suitable for determining PGDF-BB levels in healthy and patients with cancer diseases.

The envisioned biosensor was a suggested concept to study the in-situ integration of C-MEMS microelectrode with the BPE-graphene technique’s compatibility for biotechnology applications. The simplicity of electrochemical bipolar exfoliation of graphene and the process’s environmental friendliness has a high potential for other various applications, including but not limited to biofuel cells, batteries, and supercapacitors.

## 4. Conclusions

This study investigated the in-situ integration of bipolar exfoliated graphene and 3D C-MEMS microelectrodes. The SEM analysis confirmed a uniform deposition of rGO on micropillars and the support thin film electrode. The CV analysis confirmed enhanced areal capacitance after deposition of rGO, resulting from increased surface area and improved conductivity of the 3D C-MEMS electrodes after deposition of rGO. The aptasensors based on 3D C-MEMS electrodes decorated with BPE-rGO showed a high sensitivity and low limit. The achieved results suggest that bipolar electrochemistry can offer a highly efficient and environmentally friendly technology for the deposition of rGO on C-MEMS microelectrodes, which is highly promising for lab-on-chip biosensors. Furthermore, this novel integration of rGO on C-MEMS microelectrodes can be used for developing C-MEMS/rGO based biofuel cells, supercapacitors, and batteries.

## Figures and Tables

**Table 1:** The performance of recent electrochemical PDGF-BB aptasensors.

Electrode	Modification	Technique	Detection Strategy	LoD	Linear Range	Ref.
GCE	Cu-MOFs/TpBD-COFs	DPV	label-free	0.034 pg mL^−1^	0.0001–60 ng mL^−1^	[46]
	HAP-NPs	SWV	labeled: HAP-NPs	50 fg mL^−1^	0.1 pg mL^−1^–10 ng mL^−1^	[47]
C-MEMS thin film	oxygen–plasma etching	CV	label-free	7 pM	0.01–50 nM	[14]
		EIS		1.9 pM	0.005–50 nM	
Au	AuNPs	SWV	labeled: α-cyclodextrin	0.52 nM	0.52–1.52 nM	[43]
	graphene doped with silver nanoclusters	EIS	label-free	26.5 fM	32.3 fM–1.61 pM	[44]
PET/Au	BPE-rGO	DPV	label-free	0.65 pM	0.0007–20 nM	[15]
C-MEMS	BPE-rGO	CV	label-free	0.75 pM	0.001–10 nM	This work

GCE: glassy-carbon electrode; Cu-MOFs: Cu-based metal–organic frameworks; TpBD-COFs: 1,3,5-triformylphloroglucinol (Tp), benzidine (BD), covalent organic frameworks (COFs); HAP-NPs: Hydroxyapatite nanoparticles; SWV: square wave voltammetry; DVP: differential pulse voltammetry; PET: polyethylene terephthalate; EIS: electrochemical impedance spectroscopy; AuNPs: gold nanoparticles.
